# Primary Culture of Undifferentiated Pleomorphic Sarcoma: Molecular Characterization and Response to Anticancer Agents

**DOI:** 10.3390/ijms18122662

**Published:** 2017-12-08

**Authors:** Alessandro De Vita, Federica Recine, Laura Mercatali, Giacomo Miserocchi, Chiara Spadazzi, Chiara Liverani, Alberto Bongiovanni, Federica Pieri, Roberto Casadei, Nada Riva, Valentina Fausti, Dino Amadori, Toni Ibrahim

**Affiliations:** 1 Osteoncology and Rare Tumors Center, Istituto Scientifico Romagnolo per lo Studio e la Cura dei Tumori (IRST) IRCCS, Via P. Maroncelli 40, 47014 Meldola, Italy; federica.recine@irst.emr.it (F.R.); laura.mercatali@irst.emr.it (L.M.); giacomo.miserocchi@irst.emr.it (G.M.); chiara.spadazzi@irst.emr.it (C.S.); chiara.liverani@irst.emr.it (C.L.); alberto.bongiovanni@irst.emr.it (A.B.); nada.riva@irst.emr.it (N.R.); valentina.fausti@irst.emr.it (V.F.); editing@irst.emr.it (D.A.); toni.ibrahim@irst.emr.it (T.I.); 2 Pathology Unit, Morgagni-Pierantoni Hospital, Via Carlo Forlanini 34, 47121 Forlì, Italy; federica.pieri@auslromagna.it; 3 Department of Orthopedics, Istituto Ortopedico Rizzoli, University of Bologna, Via Pupilli 1, 40136 Bologna, Italy; roberto.casadei@ior.it

**Keywords:** undifferentiated pleomorphic sarcoma, malignant fibrous histiocytoma, primary culture, EMT, drug resistance, chemotherapy, eribulin

## Abstract

Undifferentiated pleomorphic sarcoma (UPS) is an aggressive mesenchymal neoplasm with no specific line of differentiation. Eribulin, a novel synthetic microtubule inhibitor, has shown anticancer activity in several tumors, including soft tissue sarcomas (STS). We investigated the molecular biology of UPS, and the mechanisms of action of this innovative microtubule-depolymerizing drug. A primary culture from a patient with UPS was established and characterized in terms of gene expression. The activity of eribulin was also compared with that of other drugs currently used for STS treatment, including trabectedin. Finally, Western blot analysis was performed to better elucidate the activity of eribulin. Our results showed an upregulation of epithelial mesenchymal transition-related genes, and a downregulation of epithelial markers. Furthermore, genes involved in chemoresistance were upregulated. Pharmacological analysis confirmed limited sensitivity to chemotherapy. Interestingly, eribulin exhibited a similar activity to that of standard treatments. Molecular analysis revealed the expression of cell cycle arrest-related and pro-apoptotic-related proteins. These findings are suggestive of aggressive behavior in UPS. Furthermore, the identification of chemoresistance-related genes could facilitate the development of innovative drugs to improve patient outcome. Overall, the results from the present study furnish a rationale for elucidating the role of eribulin for the treatment of UPS.

## 1. Introduction

Undifferentiated pleomorphic sarcoma (UPS), previously known as malignant fibrous histiocytoma (MFH), is a mesenchymal malignancy of soft tissue or bone that shows no definable line of differentiation. It is characterized by highly variable morphologic features, for the most part consisting of transition from storiform to pleomorphic areas. UPS is the fourth most common soft tissue sarcoma (STS) usually affecting elderly patients, with a slight male predominance [1]. Its incidence in children is very rare [2]. Although the majority of UPSs are high-grade lesions with a predilection for the lower extremities, the upper extremities can also be affected. The incidence of local recurrence varies between 19% and 31%, the rate of metastases is 31–35%, and the 5-year overall survival is 65–70% [3]. At present, there are no specific immunohistochemical markers available for the standard differential diagnosis, which is based on the exclusion of other entities i.e., dedifferentiated liposarcoma, pleomorphic leiomyosarcoma, pleomorphic liposarcoma, pleomorphic rhabdomyosarcoma, myxofibrosarcoma, poorly differentiated carcinoma, and melanoma [4]. The mainstay of therapy for localized disease is surgical resection with negative margins (R0 resection) combined with (neo)adjuvant treatments in selected cases. En bloc resection with 2-cm healthy tissue margins has been widely supplanted by limb- or function-sparing surgery, and amputation is only considered in selected cases [5]. Surgical resection with R0 margins remains the primary goal for retroperitoneal localized disease. However, large dimensions and the specific anatomy of lesions may make it difficult to obtain wide surgical margins, in which case, adjuvant radiotherapy may be useful [6,7]. The gold standard for metastatic disease is chemotherapy, but outcome is generally very poor. The most common sites of metastasis are the lungs (90% incidence), followed by bone (85%) and liver (1%) [8,9]. Anthracyclines are the most widely used drugs for the treatment of advanced and metastatic STS, including UPS, with a 16–27% response rate when used as single agents [10,11]. The association of both anthracyclines and ifosfamide has led to an improvement in response rates, but not in overall survival (OS) [12], and is used to obtain tumor shrinkage for clinical benefit. In other cases, a monotherapy regimen with doxorubicin is preferred, especially in a palliative setting [13]. The recent advent of novel agents with encouraging preliminary results may further improve treatment options and clinical outcomes.

**Figure 1:**
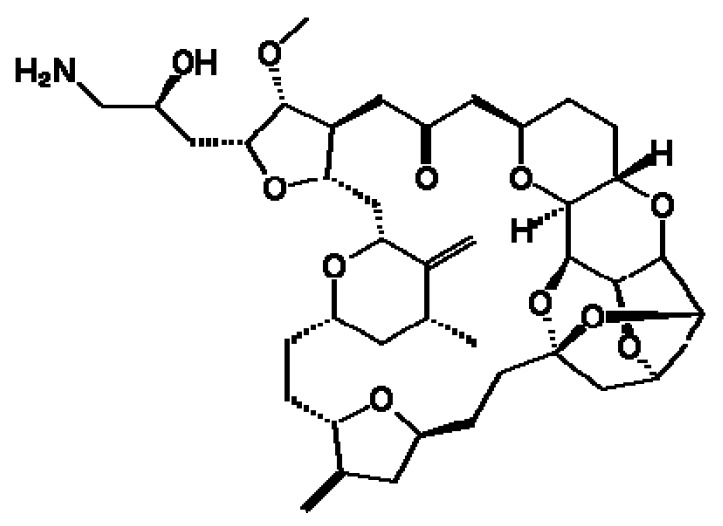
Chemical structure of eribulin.

Eribulin mesylate (Figure 1) is a novel marine-derived synthetic macrocyclic ketone analog of halichondrin B, with an innovative mechanism of action involving the inhibition of microtubule dynamics. In 2010, eribulin was approved by the U.S. Food and Drug Administration (FDA) as monotherapy for patients with advanced/metastatic breast cancer previously treated with an anthracycline or taxane [14]. Recently, eribulin was also FDA- and European Medicines Agency (EMA)-approved for the treatment of patients with inoperable liposarcoma (LPS) after the failure of anthracycline-based therapy [15]. This microtubule inhibitor drug has an antitumor activity that differs from any other microtubule-depolymerizing drug. In particular, its unique mechanism of action would seem to involve the disruption of microtubule polymerization through site-specific binding of β-tubulin, with no effect on depolymerization [16]. The consequent sequestration of β-tubulin into non-functional aggregates leads to the inhibition of microtubule dynamics, cancer cell growth- and cell cycle-arrest, and finally, apoptosis [17,18,19]. However, although several studies have demonstrated the antitumor activity of eribulin, its mechanism of action is still not clearly understood. Recent preclinical studies have shown other complex effects mediated by eribulin, including vasculature alteration, inhibition of Wnt/β-catenin signaling, suppression of migration and invasion, and reversal of epithelial–mesenchymal transition in breast cancer, adenocarcinoma, and liposarcoma [20,21,22,23]. In a non-randomized multicenter phase II study, the European Organization for Research and Treatment of Cancer (EORTC) Soft Tissue and Bone Sarcoma Group (STBSG) assessed the activity and safety of eribulin in patients with different STS histotypes, but did not report specific results on UPS [24]. The following phase III study by the same group compared the efficacy of eribulin and dacarbazine in advanced LPS and leiomyosarcoma patients [25]. However, few data are available on eribulin activity in other aggressive sarcoma subtypes known for their refractoriness to chemotherapy [26,27].

Our understanding of the molecular biology of UPS is limited, as other STS, by the small number of cell lines available [28,29]. In this regard, primary cultures offer the ideal material to study the heterogeneous biology and behavior of UPS. We established a UPS primary culture from a surgically resected specimen of a 68 year old male patient. The primary culture was characterized in terms of gene expression, and, as the disease is aggressive, we also investigated epithelial mesenchymal transition (EMT) and drug resistance-related gene expression. We compared the anticancer activity of eribulin with that of standard drugs, to elucidate the role of eribulin in UPS treatment. We also investigated the efficacy of trabectedin, a marine-derived anticancer agent that has shown to improve the clinical outcome of patients with metastatic STS, especially liposarcomas and leiomyosarcomas (referred to as l-sarcomas in the literature) [30,31]. Trabectedin was approved in 2007 by the EMA, and in 2015 by the FDA, for the treatment of advanced STS after failure of anthracyclines or for disease not amenable to treatment with these agents [32]. In addition, we explored the efficacy of dacarbazine, a treatment option for metastatic STS (including UPS), which was used as control in a phase III trial evaluating eribulin efficacy in patients with advanced liposarcoma or leiomyosarcoma [25]. Finally, we studied the molecular mechanism by which eribulin exerted its cytotoxic effect on our UPS primary culture to improve current understanding of the antitumor effect of this tubulin inhibitor.

The present work provides a valuable insight into the natural history of UPS, and sheds light on the potential role of eribulin and other drugs, including trabectedin, for the treatment of this poorly understood cancer.

## 2. Results

### 2.1. Tumor Diagnosis

**Figure 2:**
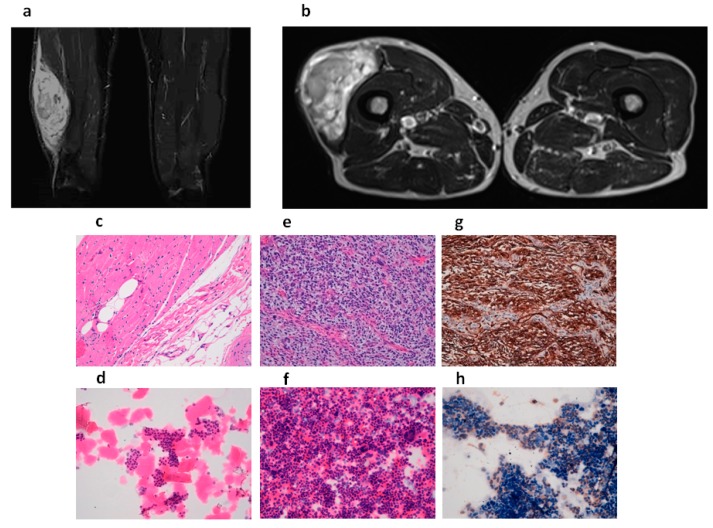
(**a**) Coronal post-contrast MRI image showing the presence of a large solid mass in the vastus lateralis of the right quadriceps femoris muscle (longitudinal diameter 20 cm). Absence of focal abnormalities in the remaining muscle–tendon structures of the thigh bilaterally; (**b**) Axial MRI image of the lesion (transverse diameter 53 mm × 88 mm) located in the middle–distal third of the quadriceps femoris muscle; (**c**) H&E staining of the surgical specimen showing the tumor-infiltrated muscle (20×). Margins were negative (R0 resection); (**d**) H&E staining of cytospun healthy cells from the patient-derived UPS primary culture (20×); (**e**) H&E staining of the surgical specimen showing undifferentiated pleomorphic sarcoma cells (light blue stroma) (20×); (**f**) H&E staining of cytospun UPS primary culture showing undifferentiated pleomorphic sarcoma cells (light blue stroma) (20×); (**g**) Immunohistochemical expression of CD34 in the tumor specimen showing undifferentiated pleomorphic sarcoma cells (brown cytoplasmic staining); (**h**) Immunohistochemical expression of CD34 in the cytospun UPS primary culture showing undifferentiated pleomorphic sarcoma cells (brown cytoplasmic staining) and cell nuclei stained with hematoxylin (blue spots).

The patient underwent an MRI scan of the right thigh, revealing the presence of a large, solid mass in the vastus lateralis of the quadriceps femoris muscle (maximum diameter 20 cm and transverse diameter around 5 cm) (Figure 2a,b). Macroscopic examination of the resected tumor mass, which weighed 630 g, showed a muscle segment of 21 cm × 10 cm × 6 cm covered by a lozenge of skin (18 cm × 8 cm). When incised, the mass had the appearance of a gelatinous, polylobulated nodule (9 cm × 10 cm) with healthy tissue margins. Microscopically, the hematoxylin and eosin (H&E) stained sections, reviewed by an experienced sarcoma pathologist, showed a muscle mass diffusely infiltrated by a tumor with spindle, markedly atypical, and pleomorphic cells with necrotic areas and several atypical mitoses (Figure 2e). Immunohistochemical analysis showed positivity for CD34 (Figure 2g) and negativity for muscular markers and S100. There was no MDM2 amplification (MDM2/Cep12 ratio of 1.0), thus, the diagnosis of dedifferentiated liposarcoma was excluded [33,34]. The diagnosis was UPS with R0 resection, and the negative margins were confirmed by H&E staining (Figure 2c).

### 2.2. Establishment of Patient-Derived Undifferentiated Pleomorphic Sarcoma (UPS) Culture

Patient-derived cells continued to grow after culture passages. Morphological analysis of the cultured cells performed by an experienced sarcoma pathologist confirmed the establishment of a UPS primary culture (Figure 2f). The proportion of UPS cells was 50%. Furthermore, the morphological evaluation of matched patient-derived cells from healthy tissue confirmed the establishment of the UPS primary culture (Figure 2d). Given that the immunohistochemical analysis of the UPS lesion revealed positivity for CD34, we evaluated the expression of the antigen in the primary culture, confirming the positivity of the cultured cells for that marker (Figure 2h).

### 2.3. Gene Expression Profile of Patient-Derived UPS Culture

**Figure 3:**
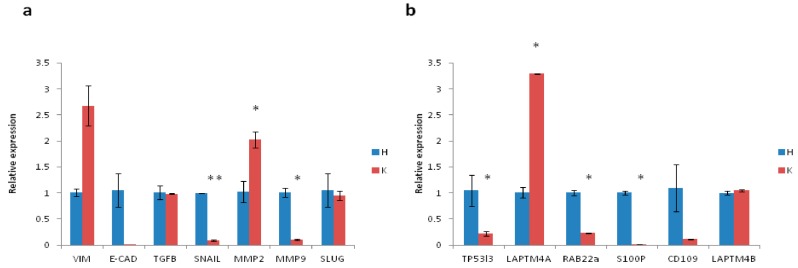
(**a**) Relative expression of EMT-related genes in the UPS primary culture (K) and matched healthy tissue (H); (**b**) Relative expression of chemoresistance-related genes and CD109 gene in the UPS primary culture and matched healthy tissue. Differences between groups were assessed by a two-tailed Student’s *t*-test, and accepted as significant * *p* < 0.05.

Several markers involved in EMT and chemoresistance were evaluated to characterize the aggressiveness of the tumor (Figure 3a,b). The expression of the mesenchymal marker *vimentin* was 2.6-fold higher than that of the control tissue, whereas *e-cadherin*, an epithelial marker, was downregulated with respect to control. *Tgf-β*, a marker involved in several tumor-associated pathways, and EMT regulators, such as *snail*, were downregulated. Expression of the matrix modifying enzyme *mmp2* was 2-fold higher that of control, while *mmp9* and the antiapoptotic, EMT-related gene *slug* were downregulated. Higher levels of some chemoresistance-related genes were also observed. In particular, the expression of *laptm4a* and *laptm4b* genes, both involved in the transport of small molecules across endosomal and lysosomal membranes [35,36], were 3.2- and 1.05-fold higher that of control, respectively.

### 2.4. Chemotherapy Assessment in UPS Primary Culture

**Figure 4:**
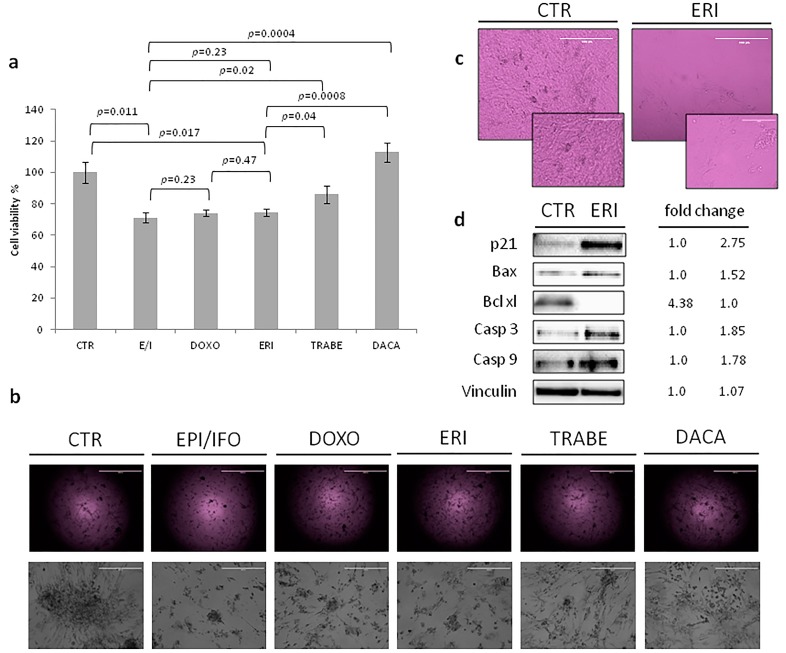
(**a**) Cytotoxicity assay in UPS primary culture exposed to the following drugs: epirubicin(EPI) plus ifosfamide (IFO), doxorubicin (DOXO), eribulin (ERI), trabectedin (TRABE), and dacarbazine (DACA). Differences between groups were assessed by a two-tailed Student’s *t*-test, and accepted as significant at *p* < 0.05; (**b**) Images of the primary culture after treatment (2× and 10× magnification, scale bar 2000 µm and 400 µm, respectively); (**c**) Morphological features observed in the primary culture after ERI treatment (20× and 40× magnification, scale bar 200 µm and 100 µm, respectively); (**d**) Western blot analysis of apoptosis-related proteins (p21, BAX, Bcl-xL; caspase-3 and caspase-9). Vinculin was used as loading control. Fold changes of band density were normalized to the band of the CTR group.

The antiproliferative activity of eribulin in the UPS primary culture was assessed by the mitochondrial reduction assay MTT (Figure 4a,b). The efficacy of eribulin (ERI) treatment was compared with that of a combination of an anthracycline (epirubicin, EPI) and ifosfamide (IFO), and an anthracycline alone (doxorubicin, DOXO), both of which represent the standard of care for unresectable or metastatic sarcomas, including UPS [12,13]. The primary culture was also exposed to the promising drug, trabectedin (TRABE), and to one of the second-line treatment options for metastatic STS, dacarbazine (DACA).

Patient-derived primary culture cells treated with the combination of EPI/IFO showed 71% survival compared to untreated controls (CTR) (EPI/IFO vs. CTR, *p* = 0.011; EPI/IFO vs. DOXO, *p* = 0.23; EPI/IFO vs. TRABE, *p* = 0.02, EPI/IFO vs. DACA, *p* = 0.0004) (Figure 4a). Treatment with DOXO resulted in 74% cell survival, 74% with ERI (ERI vs. CTR, *p* = 0.017; ERI vs. EPI/IFO, *p* = 0.23; ERI vs. DOXO *p* = 0.47; ERI vs. TRABE, *p* = 0.04; ERI vs. DACA *p* = 0.0008) and 86% with TRABE. DACA did not affect survival. Images of the UPS culture (Figure 4b) acquired after drug exposure were consistent with data obtained from the cytotoxicity assay. In particular, a similar cell confluence of EPI/IFO, DOXO and ERI was seen, while TRABE and DACA showed a higher confluence compared to the previous treatments.

### 2.5. The Activity of Eribulin in the UPS Primary Culture

Cell morphology was examined after treatment to gain a further insight into the mechanism through which eribulin exerts its anticancer activity. Morphological changes, such as rounding up and cell shrinkage, were observed after exposure to eribulin, while untreated cells continued to proliferate with a storiform pattern, and did not show these specific features (Figure 4c). We thus analyzed the expression level of some apoptosis-related proteins to determine how this microtubule-targeted drug induces its cytotoxic effect (Figure 4d). Cell cycle arrest mediated by eribulin was confirmed by the upregulation of p21, whose expression was 2.75-fold higher than that of control. Furthermore, the expression levels of pro-apoptotic protein Bax and anti-apoptotic protein Bcl-xL were evaluated to investigate the mechanisms involved in inducing apoptosis. The results showed an upregulation of Bax and downregulation of Bcl-xL, confirming the drug-mediated induction of apoptosis. Finally, since the expression of Bax was promoted by eribulin, its downstream proteins were further assessed. Caspase-3 and caspase-9 were upregulated, indicating that eribulin exerts its antitumor activity through the activation of a caspase-dependent apoptotic pathway.

## 3. Discussion

UPS, formerly known as malignant fibrous histiocytoma, was recognized for the first time as a distinct histologic STS subtype in the early 1960s [37,38]. It represents a spectrum of tumors with fibroblastic/myofibroblastic features, and no definable line of differentiation [39]. The disease manifests a wide range of histologic appearances, such as marked pleomorphism with spindle cells, multinucleated giant cells, and storiform areas [3]. UPS occurs mainly in adults, with the highest incidence rates between 50 and 70 years of age [40]. Diagnosis is based on the evaluation of hematoxylin-eosin stained sections from biopsied lesions, as specific tumor biomarkers have yet to be identified. Thus, the role of immunohistochemistry is mainly to exclude other diseases [3]. From a clinical point of view, the majority of UPS are high-grade lesions, and the incidence of local recurrence ranges between 19% and 31%. Metastases occur in 31–35% of patients, and the 5-year overall survival is 65–70% [41]. The standard therapeutic strategy for localized disease is surgery with (neo)adjuvant treatments in selected cases [5]. Chemotherapy is the standard of care in a metastatic setting, but its role is limited, and outcomes are generally poor [42]. A better understanding of the molecular background and treatment sensitivity of UPS is needed to improve the management and outcome of patients. In this regard, well-characterized cell lines could be fundamental in elucidating tumor pathophysiology, mechanisms of resistance, and the role of chemotherapy in UPS. However, given that UPS was only recently acknowledged as a distinct pathological entity, there are still no commercial UPS cell lines available [29]. Thus, patient-derived primary cultures represent the ideal experimental material to study the heterogeneous biology of UPS [43,44,45,46,47].

In the present work, cells were derived from a UPS lesion of a 68-year old male patient who underwent tumor resection. Morphological and immunohistochemical analysis confirmed the establishment of the UPS primary culture (Figure 2f,h). Gene expression analysis of the tumor specimen confirmed the mesenchymal origin of this disease. In particular, *vimentin* and *mmp2*, both mesenchymal-related genes, were upregulated, whereas the expression of *e-cadherin*, an epithelial gene, was lower than that of matched healthy tissue (Figure 3a). Furthermore, upregulation of genes involved in drug resistance, especially to anthracyclines, was observed. In this regard, the amplification of *laptm4a* and *laptm4b* genes has been reported in numerous chemoresistant tumors, including breast and gallbladder cancer [36,48]. These genes would seem to be involved in the membrane trafficking of drugs, especially anthracyclines. Thus, their overexpression could partially explain the similar antiproliferative activity of the anthracycline-based regimens (EPI/IFO and DOXO) used in our study. Further analyses are needed to elucidate the role of these genes in UPS. We also evaluated the diagnostic impact of CD109 expression, previously analyzed by our group in a series of patient-derived myxofibroscarcoma primary cultures [49]. Results showed a downregulation of *cd109* gene in this STS histotype with respect to healthy donors, indicating the potential usefulness of *cd109* in the standard differential diagnosis of MFS [49,50].

**Figure 5:**
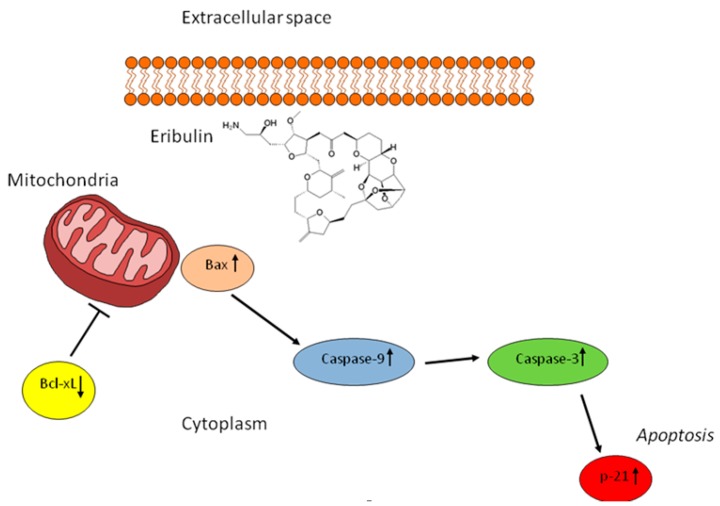
Potential mechanism of action of the cytotoxic effect of eribulin in our patient-derived UPS primary culture.

Finally, in order to elucidate the role of chemotherapy in UPS, we exposed the primary culture to different drugs currently used for the treatment of advanced STS. The activity of novel drugs, such as trabectedin and eribulin, the latter under investigation for use in STS, was also assessed. Results revealed a similar activity of doxorubicin alone, the epirubicin–ifosfamide combination, and eribulin alone. Although trabectedin showed a lower antiproliferative effect than the above drugs, it nevertheless induced a cytotoxic effect, confirming previous reports of its antitumor activity in UPS [51,52]. Dacarbazine did not affect cell survival. The significant anticancer activity of eribulin in the primary culture with respect to the first-line ifosfamide–epirubicin combination, prompted us to analyze the mechanism of action through which eribulin exerts its antitumor activity in UPS. Morphological changes in the cell culture, such as rounding up and cell shrinkage, were detected after eribulin treatment with respect to control cells, which were characterized by a storiform pattern (Figure 4b,c). We thus analyzed apoptosis-related proteins to better understand the cytotoxic activity mediated by this new interfering microtubule. Results indicated that eribulin exerted its antiproliferative activity through the activation of p21 and Bax, and the suppression of Bcl-xL. The upregulation of the 2 pro-apoptotic proteins led to the collapse of mitochondrial transmembrane potential. Finally, investigation of the downstream proteins caspase-3 and caspase-9 showed that eribulin enhanced their expression. These data suggest that eribulin induces cell cycle arrest and cell death programming through a caspase-dependent mitochondrial pathway (Figure 5).

## 4. Experimental Section

### 4.1. Ethics Statement

Briefly, the tumor mass was harvested intraoperatively from a patient undergoing surgical resection, as reported in the Results section. The study was approved by IRST-Area Vasta Romagna Ethics Committee, approval no. 4751, 31 July 2015, and all the procedures were performed following Good Clinical Practice and in accordance with the principles laid down in the Helsinki declaration. The patient gave written informed consent to take part in the study.

### 4.2. Patient History

A 68 year old male presented with a solitary, rapidly growing lesion in his right thigh. His past medical history was unremarkable. An MRI scan showed a large solid lesion in the quadriceps femoris muscle of the limb (longitudinal diameter 20 cm and transverse diameters 53 mm × 88 mm). A biopsy revealed the presence of a malignant mesenchymal tumor with poorly differentiated myxoid aspects compatible with undifferentiated pleomorphic sarcoma. A full-body CT scan was negative. The patient underwent surgical resection of the lesion, the pathology report describing a nodular lesion (9 cm × 10 cm) from high-grade undifferentiated pleomorphic sarcoma with wide disease-free margins. As there was no evidence of metastatic disease post-surgery, adjuvant radiotherapy was administered. Chest CT re-staging after radiation treatment revealed the presence of a single lung lesion suggestive of metastasis, and subsequently confirmed as such by an FDG PET/CT scan (Siemens Healthcare Ltd., Milan, Italy). The patient recently underwent metastasectomy.

### 4.3. Histological and Immunohistochemical Diagnosis

The surgical specimen was selected and analyzed by an experienced sarcoma pathologist, and processed within 3 h of surgical resection. Normal tissue comprising thigh muscle and adipose tissue and blood vessels obtained by tumor R0 resection was used as study control (Figure 2c). Differential diagnosis was performed by hematoxylin and eosin (H&E, Sigma Aldrich, St. Louis, MO, USA) staining. Briefly, the surgical material was washed twice with sterile phosphate buffered saline (PBS), paraffin-embedded in a cryomold and then frozen at −80 °C. The frozen tissue blocks were then sectioned into 5 µm-thick slices using a microtome, after which the sections were hydrated and stained with hematoxylin (Sigma Aldrich, St. Louis, MO, USA) and eosin (Sigma-Aldrich), according to the manufacturer’s instructions. Finally, the stained sections were washed three times with PBS 1×, mounted with Cytoseal™ XYL (Thermo Scientific™ Richard-Allan Scientific™, San Diego, CA, USA) mounting media, covered with a coverslip, and analyzed. CD34 determination was performed by immunohistochemical staining. Briefly, 5 µm-thick sections cut from paraffin-embedded tissue were deparaffinized with xylene for 1 h, after which they were rehydrated and incubated with antigen retrieval solution in a water bath at 98.5 °C for 30 min. The sections were cooled for 20 min, incubated for 10 min with 3% hydrogen peroxide solution, and washed twice with demineralized water. They were then incubated with 3% bovine serum albumin in PBS for 20 min, and incubated with CD34 antibody (Abcam^®^, Cambridge, UK) diluted 1:200 at room temperature for one hour. CD34 antibody was detected by immunoperoxidase techniques using the streptavidin–biotin–peroxidase complex (ABC) method [53]. Cell nuclei were counterstained with hematoxylin (Sigma Aldrich). The slides were mounted with Cytoseal™ XYL (Thermo Scientific™ Richard-Allan Scientific™) mounting media, covered with a coverslip, and analyzed. Finally, MDM2 gene amplification was assessed by FISH analysis (Vysis MDM2/CEP 12 FISH Probe Kit, Abbott Park, IL, USA) to exclude a diagnosis of dedifferentiated liposarcoma.

### 4.4. Establishment of UPS Primary Cell Culture

A patient-derived UPS primary cell culture was established from surgical tissue. The tumor specimens were washed twice in PBS, and shredded into 1–2 mm^3^ pieces with surgical scalpels. The obtained pieces were then incubated with a PBS solution of 2 mg/mL collagenase type I (Millipore Corporation, Billerica, MA, USA) at 37 °C in stirring conditions for 15 min, after which the sample was stored overnight at room temperature. Collagenase digestion was then blocked by adding DMEM supplemented with 10% fetal bovine serum, 1% glutamine, and 10% penicillin/streptomycin, after which cells were isolated from the aggregates using a 100 µm sterile filter (CellTrics, Partec, Münster, Germany). Cells were counted and seeded in standard monolayer cultures at a density of 80,000 cells/cm^2^, and maintained in complete DMEM medium at 37 °C in a 5% CO_2_ atmosphere. All the experiments were performed with the use of low-passage primary cultures, and analyzed by an experienced sarcoma pathologist.

### 4.5. Histological and Immunohistochemical Analysis of UPS Primary Culture

Hematoxylin and eosin (H&E) staining was performed to evaluate the morphological features and distribution of cells of the patient-derived UPS culture. Cells (100,000) were cytospun to glass slides, fixed in acetone for 10 min, and then in chloroform for 5 min, and stored at −20 °C. Finally, the slides were thawed, hydrated, and stained with H&E following the manufacturer’s instructions. The stained slides were analyzed with an optical Zeiss Axioskop microscope (Carl Zeiss, Gottïngen, Germany) equipped with a Polaroid camera. CD34 immunohistochemical analysis was performed on the cytological slides following the protocol reported in the Histological and Immunohistochemical Diagnosis section. The stained slides were analyzed with an optical Zeiss Axioskop microscope equipped with a Polaroid camera. 

### 4.6. Gene Expression Analysis

RNA extraction was carried out using the cells isolated from tissue specimens without being cultured in vitro, in order to avoid molecular changes. Briefly, mRNA isolation was performed using TRIzol Reagent (Invitrogen, Carlsbad, CA, USA) following the manufacturer’s instructions. iScript cDNA Synthesis Kit (BioRad, Hercules, CA, USA) was used to reverse transcribe 500 ng of extracted RNA. Gene expression analysis was then carried out by Real-Time PCR using a 7500 Real-Time PCR System (Applied Biosystems, Foster City, CA, USA). Amplification was performed in a total volume of 20 µL containing 2× Taqman Universal PCR Master Mix (Applied Biosystems) and 2 µL of cDNA. The following markers were analyzed: *vimentin*, *e-cadherin*, *tgf-β*, *snail*, *mmp2*, *mmp9*, and *slug*. *Actb* and *hprt* were used as reference genes. The resulting amount of the transcripts was normalized to the reference genes with the 2(-delta delta C(T)) method. We used SYBR Select Master Mix (Applied Biosystems) with 2 µL of cDNA for *tp53i3*, *laptm4a*, *rab22a*, *s100p*, *cd109*, and *laptm4b* analysis. *Actb* and *gapdh* were used as reference genes.

### 4.7. Drug Testing

For drug assessment, 10,000 cells/well were seeded in 96-well plates. Cells were allowed to recover for 3 days, and were then treated. Drug regimens were selected according to the plasma peak concentration of each drug from pharmacokinetic clinical data; epirubicin 2 µg/mL (Accord Healthcare Italia Ltd., Milan, Italy) plus ifosfamide 100 µM (Baxter Ltd., Rome, Italy) [54,55,56,57,58], doxorubicin 4 µg/mL (Accord Healthcare Italia Ltd., Milan, Italy) [57], 371 ng/mL eribulin (Eisai Ltd., Milan, Italy) [22,59], trabectedin 17 ng/mL (PharmaMar Ltd., Milan, Italy) [60], and dacarbazine 8 ug/mL (Medac Pharma Ltd., Rome, Italy) [61]. Survival percentages were assessed, as previously reported [62], by MMT assay (Sigma Aldrich) after drug exposure for 72 h. Experiments were performed twice.

### 4.8. Protein Expression Analysis

Patient-derived cell cultures were exposed to eribulin at the human plasma peak concentration, as reported in the Drug Testing section. After 72 h, cells were trypsinized, immediately frozen with liquid nitrogen, and stored at −80 °C. Proteins were then extracted using a RIPA buffer with 10% PMSF, 1% HALT phosphatase inhibitor cocktail, and 0.5% protease inhibitor cocktail. The cell suspension was centrifuged at 15,000 rpm for 20 min at 4 °C and protein contents were determined using a BCA protein assay kit (Pierce™ BCA Protein Assay Kit, Thermo Scientific, Waltham, MA, USA). An equal amount of protein from each sample was separated on Criterion™ Precast Gel Tris-HCl (Biorad, Hercules, CA, USA) and transferred to polyvinylidene fluoride membranes (Millipore Corporation). The membranes were blocked for 2 h with a solution containing 5% fat-free milk PBS with 0.1% Tween 20 (Sigma Aldrich, St. Louis, MO, USA) at room temperature, and incubated overnight at 4 °C with each of the following antibodies: anti-p21 (1:2000 Cell Signaling, Danvers, MA, USA), anti-Bax (1:1000 Cell Signaling), anti-Bcl-xL (1:1000 Cell Signaling), anti-caspase-3 (1:1000 Cell Signaling), anti-caspase-9 (1:1000 Cell Signaling), and anti-vinculin (1:1000 Thermo Scientific). After washing, the membranes were incubated for 1 h at room temperature with horseradish peroxidase-conjugated secondary antibody. Densitometric analysis of proteins was performed on Western blot with Quantity One software version 4.6.9 (BioRad, Hercules, CA, USA).

### 4.9. Statistical Analysis

Three independent replicates were performed for each experiment. Data are presented as mean ± standard deviation (SD), or mean ± standard error (SE), as stated, with *n* indicating the number of replicates. Differences between groups were assessed by a two-tailed Student’s *t*-test, and accepted as significant at *p* < 0.05.

## 5. Conclusions

In conclusion, the results from the present study highlight the potential importance of using patient-derived primary cultures to study the tumor heterogeneity and clinical diversity of UPS. They also increase our understanding of the molecular background of this STS histotype, and could contribute to the development of new therapeutic strategies. Finally, our investigation of the mechanism of action of the promising microtubule-depolymerizing drug, eribulin, provides a rationale for a more in-depth evaluation of its role in the treatment of this mesenchymal malignancy.

## References

[1] Fletcher C.D.M., Bridge J.A., Hogendoorn P., Mertens F. (2013). WHO Classification of Tumours of Soft Tissue Sarcoma and Bone.

[2] Alaggio R., Collini P., Randall R.L., Barnette P., Million L., Coffin C.M. (2010). Undifferentiated high-grade pleomorphic sarcomas in children: A clinicopathologic study of 10 cases and review of literature. Pediatr. Dev. Pathol..

[3] Goldblum J.R. (2014). An approach to pleomorphic sarcomas: Can we subclassify, and does it matter?. Mod. Pathol..

[4] Nascimento A.F., Raut C.P. (2008). Diagnosis and management of pleomorphic sarcomas (so-called “MFH”) in adults. J. Surg. Oncol..

[5] Saponara M., Stacchiotti S., Casali P.G., Gronchi A. (2017). (Neo)adjuvant treatment in localised soft tissue sarcoma: The unsolved affair. Eur. J. Cancer.

[6] Nussbaum D.P., Rushing C.N., Lane W.O., Cardona D.M., Kirsch D.G., Peterson B.L., Blazer D.G. (2016). Preoperative or postoperative radiotherapy versus surgery alone for retroperitoneal sarcoma: A case-control, propensity score-matched analysis of a nationwide clinical oncology database. Lancet Oncol..

[7] McBride S.M., Raut C.P., Lapidus M., Devlin P.M., Marcus K.J., Bertagnolli M., George S., Baldini E.H. (2013). Locoregional recurrence after preoperative radiation therapy for retroperitoneal sarcoma: Adverse impact of multifocal disease and potential implications of dose escalation. Ann. Surg. Oncol..

[8] Kearney M.M., Soule E.H., Ivins J.C. (1980). Malignant fibrous histiocytoma: A retrospective study of 167 cases. Cancer.

[9] Pezzi C.M., Rawlings M.S., Esgro J.J., Pollock R.E., Romsdahl M.M. (1992). Prognostic factors in 227 patients with malignant fibrous histiocytoma. Cancer.

[10] Schoenfeld D., Rosenbaum C., Horton J. (1982). A comparison of Adriamycin versus vincristine and Adriamycin and cyclophosphamide for advanced sarcoma. Cancer.

[11] Bramwell V.H.C., Anderson D., Charette M.L. (2000). Doxorubicin-based chemotherapy for the palliative treatment of adult patients with locally advanced or metastatic soft-tissue sarcoma: A meta-analysis and clinical practice guideline. Sarcoma.

[12] Judson I., Verweij J., Gelderblom H., Hartmann J.T., Schöffski P., Blay J.Y., Kerst J.M., Sufliarsky J., Whelan J., Hohenberger P. (2014). Doxorubicin alone versus intensified doxorubicin plus ifosfamide for first-line treatment of advanced or metastatic soft-tissue sarcoma: A randomised controlled phase 3 trial. Lancet Oncol..

[13] Gronchi A., Stacchiotti S., Verderio P., Ferrari S., Martin Broto J., Lopez-Pousa A., Llombart-Bosch A., Dei Tos A.P., Collini P., Jurado J.C. (2016). Short, full-dose adjuvant chemotherapy (CT) in high-risk adult soft tissue sarcomas (STS): Long-term follow-up of a randomized clinical trial from the Italian Sarcoma Group and the Spanish Sarcoma Group. Ann. Oncol..

[14] Swami U., Shah U., Goel S. (2015). Eribulin in cancer treatment. Mar. Drugs.

[15] Thomas C., Movva S. (2016). Eribulin in the management of inoperable soft-tissue sarcoma: Patient selection and survival. Onco Targets Ther..

[16] Smith J.A., Wilson L., Azarenko O., Zhu X., Lewis B.M., Littlefield B.A., Jordan M.A. (2010). Eribulin binds at microtubule ends to a single site on tubulin to suppress dynamic instability. Biochemistry.

[17] Jordan M.A., Kamath K., Manna T., Okouneva T., Miller H.P., Davis C., Littlefield B.A., Wilson L. (2005). The primary antimitotic mechanism of action of the synthetic halichondrin E7389 is suppression of microtubule growth. Mol. Cancer Ther..

[18] Kuznetsov G., Towle M.J., Cheng H., Kawamura T., TenDyke K., Liu D., Kishi Y., Yu M.J., Littlefield B.A. (2004). Induction of morphological and biochemical apoptosis following prolonged mitotic blockage by halichondrin B macrocyclic ketone analog E7389. Cancer Res..

[19] Towle M.J., Salvato K.A., Budrow J., Wels B.F., Kuznetsov G., Aalfs K.K., Welsh S., Zheng W., Seletsky B.M., Palme M.H. (2001). In vitro and in vivo anticancer activities of synthetic macrocyclic ketone analogues of halichondrin B. Cancer Res..

[20] Funahashi Y., Okamoto K., Adachi Y., Semba T., Uesugi M., Ozawa Y., Tohyama O., Uehara T., Kimura T., Watanabe H. (2014). Eribulin mesylate reduces tumor microenvironment abnormality by vascular remodeling in preclinical human breast cancer models. Cancer Sci..

[21] Suzuki H., Hirata Y., Suzuki N., Ihara S., Sakitani K., Kobayashi Y., Kinoshita H., Hayakawa Y., Yamada A., Watabe H. (2015). Characterization of a new small bowel adenocarcinoma cell line and screening of anti-cancer drug against small bowel adenocarcinoma. Am. J. Pathol..

[22] De Vita A., Miserocchi G., Recine F., Mercatali L., Pieri F., Medri L., Bongiovanni A., Cavaliere D., Liverani C., Spadazzi C. (2016). Activity of eribulin in a primary culture of well-differentiated/dedifferentiated adipocytic sarcoma. Molecules.

[23] Yoshida T., Ozawa Y., Kimura T., Sato Y., Kuznetsov G., Xu S., Uesugi M., Agoulnik S., Taylor N., Funahashi Y. (2014). Eribulin mesilate suppresses experimental metastasis of breast cancer cells by reversing phenotype from epithelial-mesenchymal transition (EMT) to mesenchymal-epithelial transition (MET) states. Br. J. Cancer.

[24] Schöffski P., Ray-Coquard I.L., Cioffi A., Bui N.B., Bauer S., Hartmann J.T., Krarup-Hansen A., Grünwald V., Sciot R., Dumez H. (2011). Activity of eribulin mesylate in patients with soft-tissue sarcoma: A phase 2 study in four independent histological subtypes. Lancet Oncol..

[25] Schöffski P., Chawla S., Maki R.G., Italiano A., Gelderblom H., Choy E., Grignani G., Camargo V., Bauer S., Rha S.Y. (2016). Eribulin versus dacarbazine in previously treated patients with advanced liposarcoma or leiomyosarcoma: A randomised, open-label, multicentre, phase 3 trial. Lancet.

[26] Kawai A., Araki N., Naito Y., Ozaki T., Sugiura H., Yazawa Y., Morioka H., Matsumine A., Saito K., Asami S. (2017). Phase 2 study of eribulin in patients with previously treated advanced or metastatic soft tissue sarcoma. Jpn. J. Clin. Oncol..

[27] Emambux S., Kind M., Le Loarer F., Toulmonde M., Stoeckle E., Italiano A. (2017). Clinical activity of eribulin in advanced desmoplastic small round-cell tumor. Anticancer Drugs.

[28] Nishio J., Iwasaki H., Ishiguro M., Ohjimi Y., Nishimura N., Koga T., Kawarabayashi T., Kaneko Y., Kikuchi M. (2003). Establishment of a new human malignant fibrous histiocytoma cell line, FU-MFH-1: Cytogenetic characterization by comparative genomic hybridization and fluorescence in situ hybridization. Cancer Genet. Cytogenet..

[29] Pan X., Yoshida A., Kawai A., Kondo T. (2016). Current status of publicly available sarcoma cell lines for use in proteomic studies. Expert Rev. Proteom..

[30] Demetri G.D., von Mehren M., Jones R.L., Jones R.L., Hensley M.L., Schuetze S.M., Staddon A., Milhem M., Elias A., Ganjoo K. (2016). Efficacy and safety of trabectedin or dacarbazine for metastatic liposarcoma or leiomyosarcoma after failure of conventional chemotherapy: Results of a phase III randomized multicenter clinical trial. J. Clin. Oncol..

[31] Demetri G.D., Chawla S.P., von Mehren M., Ritch P., Baker L.H., Blay J.Y., Hande K.R., Keohan M.L., Samuels B.L., Schuetze S. (2009). Efficacy and safety of trabectedin in patients with advanced or metastatic liposarcoma or leiomyosarcoma after failure of prior anthracyclines and ifosfamide: Results of a randomized phase II study of two different schedules. J. Clin. Oncol..

[32] Recine F., Bongiovanni A., Riva N., Fausti V., De Vita A., Mercatali L., Liverani C., Miserocchi G., Amadori D., Ibrahim T. (2017). Update on the role of trabectedin in the treatment of intractable soft tissue sarcomas. Onco Targets Ther..

[33] Weaver J., Downs-Kelly E., Goldblum J.R., Turner S., Kulkarni S., Tubbs R.R., Rubin B.P., Skacel M. (2008). Fluorescence in situ hybridization for MDM2 gene amplification as a diagnostic tool in lipomatous neoplasms. Mod. Pathol..

[34] De Vita A., Mercatali L., Recine F., Pieri F., Riva N., Bongiovanni A., Liverani C., Spadazzi C., Miserocchi G., Amadori D. (2016). Current classification, treatment options, and new perspectives in the management of adipocytic sarcomas. Onco Targets Ther..

[35] Hogue D.L., Nash C., Ling V., Hobman T.C. (2002). Lysosome-associated protein transmembrane 4 alpha (LAPTM4 alpha) requires two tandemly arranged tyrosine-based signals for sorting to lysosomes. Biochem. J..

[36] Li Y., Zou L., Li Q., Haibe-Kains B., Tian R., Li Y., Desmedt C., Sotiriou C., Szallasi Z., Iglehart J.D. (2010). Amplification of LAPTM4B and YWHAZ contributes to chemotherapy resistance and recurrence of breast cancer. Nat. Med..

[37] Ozzello L., Stout A.P., Murray M.R. (1963). Cultural characteristics of malignant histiocytomas and fibrous xanthomas. Cancer.

[38] O’Brien J.E., Stout A.P. (1964). Malignant fibrous xanthomas. Cancer.

[39] Montgomery E., Fisher C. (2001). Myofibroblastic differentiation in malignant fibrous histiocytoma (pleomorphic myofibrosarcoma): A clinicopathological study. Histopathology.

[40] Weiss S.W., Enzinger F.M. (1978). Malignant fibrous histiocytoma: An analysis of 200 cases. Cancer.

[41] Belal A., Kandil A., Allam A., Khafaga Y., El-Husseiny G., El-Enbaby A., Memon M., Younge D., Moreau P., Gray A. (2002). Malignant fibrous histiocytoma: A retrospective study of 109 cases. Am. J. Clin. Oncol..

[42] Adrianzen Herrera D.A., Kuk D., Keohan M.L., Dickson M.A., D’Angelo S.P., Ping C., Agaram N.P., Antonescu C.R., Hameed M., Tap W.D. (2016). Outcomes of systemic therapy for patients with metastatic undifferentiated pleomorphic sarcoma (UPS). J. Clin. Oncol..

[43] Miserocchi G., Mercatali L., Liverani C., De Vita A., Spadazzi C., Pieri F., Bongiovanni A., Recine F., Amadori D., Ibrahim T. (2017). Management and potentialities of primary cancer cultures in preclinical and translational studies. J. Transl. Med..

[44] Igarashi K., Kawaguchi K., Kiyuna T., Murakami T., Miwa S., Nelson S.D., Dry S.M., Li Y., Singh A.S., Kimura H. (2017). Efficacy in vitro of caffeine and valproic acid on patient-derived undifferentiated pleomorphic sarcoma and rhabdomyosarcoma cell lines. Anticancer Res..

[45] Salawu A., Fernando M., Hughes D., Reed M.W., Woll P., Greaves C., Day C., Alhajimohammed M., Sisley K. (2016). Establishment and molecular characterization of seven novel soft-tissue sarcoma cell lines. Br. J. Cancer.

[46] Schlott T., Taubert H., Fayyazi A., Schweyer S., Bartel F., Korabiowska M., Brinck U. (2004). Analysis of central regulatory pathways in p53-deficient primary cultures of malignant fibrous histiocytoma exposed to ifosfamide. Anticancer Res..

[47] Igarashi K., Kawaguchi K., Murakami T., Kiyuna T., Miyake K., Yamamoto N., Hayashi K., Kimura H., Nelson S.D., Dry S.M. (2017). A novel anionic-phosphate-platinum complex effectively targets an undifferentiated pleomorphic sarcoma better than cisplatinum and doxorubicin in a patient-derived orthotopic xenograft (PDOX). Oncotarget.

[48] Zhou L., He X.D., Yu J.C., Zhou R.L., Shan Y., Rui J.A. (2011). Overexpression of LAPTM4B-35 attenuates epirubucin induced apoptosis of gallbladder carcinoma GBC-SD cells. Surgery.

[49] De Vita A., Recine F., Mercatali L., Miserocchi G., Liverani L., Spadazzi L., Casadei R., Bongiovanni A., Pieri F., Riva N. (2017). Myxofibrosarcoma primary cultures: Molecular and pharmacological profile. Ther. Adv. Med. Oncol..

[50] Emori M., Tsukahara T., Murata K., Sugita S., Sonoda T., Kaya M., Soma T., Sasaki M., Nagoya S., Hasegawa T. (2015). Prognostic impact of CD109 expression in myxofibrosarcoma. J. Surg. Oncol..

[51] Khalifa J., Ouali M., Chaltiel L., Le Guellec S., Le Cesne A., Blay J.Y., Cousin P., Chaigneau L., Bompas E., Piperno-Neumann S. (2015). Efficacy of trabectedin in malignant solitary fibrous tumors: A retrospective analysis from the French Sarcoma Group. BMC Cancer.

[52] Le Cesne A., Cresta S., Maki R.G., Blay J.Y., Verweij J., Poveda A., Casali P.G., Balaña C., Schöffski P., Grosso F. (2012). A retrospective analysis of antitumour activity with trabectedin in translocation-related sarcomas. Eur. J. Cancer.

[53] Sabattini E., Bisgaard K., Ascani S., Poggi S., Piccioli M., Ceccarelli C., Pieri F., Fraternali-Orcioni G., Pileri S.A. (1998). Envision Plus: A new immunohistochemical method of choice for diagnostics and research. Critical comparison with the APAAP, ChemMate CSA, LABC, and SABC techniques. J. Clin. Pathol..

[54] Highley M.S., Momerency G., Sawyers D., de Bruijn E.A., Prenen H., Guetens G., de Boeck G., van Oosterom A.T., Mansi J.L., Blake P.R. (2015). The neurotoxicity and pharmacokinetics of oral ifosfamide. J. Anal. Oncol..

[55] Cerny T., Leyvraz S., von Briel T., Küpfer A., Schaad R., Schmitz S.F., Honegger P., Sessa C., Brunner J., Boddy A.V. (1999). Saturable metabolism of continuous high-dose ifosfamide with mesna and GM-CSF: A pharmacokinetic study in advanced sarcoma patients. Swiss Group for Clinical Cancer Research (SAKK). Ann. Oncol..

[56] Fogli S., Danesi R., Gennari A., Donati S., Conte P.F., del Tacca M. (2002). Gemcitabine, epirubicin and paclitaxel: Pharmacokinetic and pharmacodynamic interactions in advanced breast cancer. Ann. Oncol..

[57] Robert J., Vrignaud P., Nguyen-Ngoc T., Iliadis A., Mauriac L., Hurteloup P. (1985). Comparative pharmacokinetics and metabolism of doxorubicin and epirubicin in patients with metastatic breast cancer. Cancer Treat. Rep..

[58] Danesi R., Innocenti F., Fogli S., Gennari A., Baldini E., di Paolo A., Salvadori B., Bocci G., Conte P.F., Del Tacca M. (2002). Pharmacokinetics and pharmacodynamics of combination chemotherapy with paclitaxel and epirubicinin breast cancer patients. Br. J. Clin. Pharmacol..

[59] Devriese L.A., Witteveen P.E., Wanders J., Law K., Edwards G., Reyderman L., Copalu W., Peng F., Marchetti S., Beijnen J.H. (2013). Pharmacokinetics of eribulin mesylate in patients with solid tumours receiving repeated oral rifampicin. Br. J. Clin. Pharmacol..

[60] Van Kesteren C.H., de Vooght M.M., López-Lázaro L., Mathôt R.A., Schellens J.H., Jimeno J.M., Beijnen J.H. (2003). Yondelis (trabectedin, ET-743): The development of an anticancer agent of marine origin. Anticancer Drugs.

[61] Brendel E., Ludwig M., Lathia C., Robert C., Ropert S., Soria J.C., Armand J.P. (2011). Pharmacokinetic results of phase I trial of sorafenib in combination with dacarbazine in patients with advanced solid tumors. Cancer Chemother. Pharmacol..

[62] Mercatali L., Spadazzi C., Miserocchi G., Liverani C., De Vita A., Bongiovanni A., Recine F., Amadori D., Ibrahim T. (2016). The effect of everolimus in an in vitro model of triple negative breast cancer and osteoclasts. Int. J. Mol. Sci..

